# Polyhydroxyalkanoates Synthesized by *Aeromonas* Species: Trends and Challenges

**DOI:** 10.3390/polym11081328

**Published:** 2019-08-09

**Authors:** Justyna Możejko-Ciesielska, Paulina Marciniak, Karolina Szacherska

**Affiliations:** Department of Microbiology and Mycology, Faculty of Biology and Biotechnology, University of Warmia and Mazury in Olsztyn, Oczapowskiego 1A, 10719 Olsztyn, Poland

**Keywords:** *Aeromonas* spp., biodegradable polymers, biopolymers, polyhydroxyalkanoates

## Abstract

The negative effects of petrochemical-derived plastics on the global environment and depletion of global fossil fuel supplies have paved the way for exploring new technologies for the production of bioplastics. Polyhydroxyalkanoates (PHAs) are considered an alternative for synthetic polymers because of their biodegradability, biocompatibility, and non-toxicity. Many bacteria have been reported to have the ability to synthesize PHAs. Among them, the *Aeromonas* species seem to be ideal hosts for the industrial production of these biopolyesters due to their robust growth, simple growth requirements, their ability for the synthesis of homopolymers, co-polymers, and terpolymers with unique material properties. Some *Aeromonas* strains were able to produce PHAs in satisfactory amounts from simple carbon sources. Efforts have been made to use genetically modified *Aeromonas* strains for enhanced PHAs and to obtain bacteria with modified compositions and improved properties. This review discusses the current state of knowledge of polyhydroxyalkanoates synthesized by *Aeromonas* species, with a special focus on their potential, challenges, and progress in PHA synthesis.

## 1. Introduction

In Europe, 51 million tonnes of plastics are produced every year. In the USA, only 8% of plastics were recycled in 2010. It is estimated that the recycling of petroleum materials does not exceed 50% [1]. Problems with the utilization of wastes, as well as environmental pollution, are reasons to search for other substances, which will be less harmful to the environment and could replace petroleum-derived polymers. Scientists are looking for biodegradable polymers that can be totally degraded, and are not harmful for the environment.

Biodegradable polymers are classified into four groups, depending on their composition. Biopolymers like cellulose, lignin, natural rubber, or polyhydroxyalkanoates belong to the first group and are synthesized by microorganisms. The second group contains polymers produced by the polymerization of monomers that either exist in nature or are derived from materials that exist in nature (polylactide, polytrimethylene glycol). The third group consists of a mixture of monomers from renewable resources with synthetic monomers (soy-based urethanes). The fourth group has blends of renewable resources and petrochemical-derived components (starch and polyvinyl alcohol) [2]. However, two of the most promising biopolymers that have unique properties are polylactide (PLA) and polyhydroxyalkanaotes (PHAs). PLA has gained much attention as a potential alternative to existing materials, while PHAs have been considered in recent years as bio-based and biodegradable materials without waste, which are recycled to CO_2_ and water.

**Figure 1:**
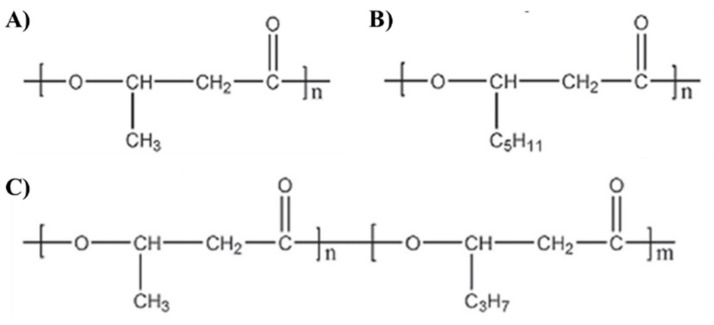
Chemical formulae of polyhydroxyalkanaotes (PHAs). (**A**) P(3HB); (**B**) P(3HO), (**C**) P(3HB-co-3HHx) [4].

PHAs are a class of linear polyesters of hydroxyalkanoic acids—the carboxylate group of one monomer is connected with the hydroxyl group of the neighbouring monomer by an ester bond. The chemical structure of PHAs is presented in Figure 1. PHAs are divided into two groups, depending on the number of carbon atoms in the monomeric units. Short-chain-length PHAs (scl-PHAs) contain from three to five carbon atoms in their monomers, for example, poly(3-hydroxybutyrate) (P(3HB)), poly(3-hydroxyvalerate) (P(3HV)), or poly(3-hydroxybutyrate-co-3-hydrovalerate) copolymer (P(3HB-co-3HV)). Medium-chain-length PHAs (mcl-PHAs) consist of six and more carbon atoms in each unit. Belonging to this group are, for example, poly(3-hydroxyhexanoate) (P(3HHx)), poly(3-hydroxyoctanoate) (P(3HO)), and poly(3-hydroxyhexanoate-co-3-hydroxyoctanoate) copolymer (P(3HHx-co-3HO)). These two classes differ mainly due to the substrate specificity of PHA synthases, which play a crucial role in the PHA polymerization process, using (R)-3-hydroxyacyl-CoA as a substrate for PHAs synthesis. For example, PHA synthase from *Aeromonas caviae* is capable of synthesizing P(3HB-*co*-3HHx) because it possesses a high affinity for both 3HB and 3HHx units, unlike other PHA synthases, which show an affinity only for the 3HB unit [3].

PHAs are polyesters synthesized intracellularly by microorganisms and stored as reserve materials, when a carbon source is present and nutrients like phosphorus, nitrogen, or magnesium are present in limited concentrations. Bacteria living in different environments are able to biosynthesize PHAs. These organisms have been isolated from sludge, water sediments, marine water, soil, and even from extreme environments such as Antarctica [5]. Today, over 90 genera of microorganisms have been reported to accumulate these biopolymers and synthesize about 150 different types of monomer constituents, which possess straight, branched, saturated, unsaturated, and aromatic structures, that provide different properties [6]. PHAs were initially used to make everyday articles like shampoo bottles, but over the last years, applications have increased both in variety and specialization. Being biodegradable, biocompatible, thermoplastic, and non-toxic, PHAs could be used in the packaging industry, biomedicine, or agriculture. During the last decades, a great effort has been made to apply them in the medical field, such as in the drug delivery system, stents production, management of wounds, cardiovascular system devices, and orthopedics [7]. A large number of animal trials have been performed to evaluate their useful properties and applicability. Recent progress in PHA applications has been comprehensively reviewed by Butt et al. [8].

Members of the *Aeromonas* genera showed some advantages for the production of polyhydroxyalkanoates, especially scl-mcl-PHAs copolymers. Furthermore, their robust growth and simple substrate requirements make these bacteria potential hosts for the industrial production of biopolyesters [9]. P(3HB-3HHx) was produced by *Aeromonas hydrophila* on a large scale. Tsinghua University (China), in collaboration with Guangdong Jiangmen Center for Biotech Development (China), KAIST (Korea), and Procter & Gamble (USA), developed a fermentation strategy in a 20,000 L bioreactor [10]. The extracted and purified biopolymer was used to make flushables, nonwovens, binders, flexible packaging, thermoformed articles, synthetic paper, and medical devices [11].

This review includes discussions of the potential, challenges, and progress in PHA synthesis by the *Aeromonas* species. It summarizes recent trends towards PHA production as an effort to provide direction towards eco-sustainable development. This review focuses on the development of fermentation strategies, PHA synthesis by wild and genetically engineered *Aeromonas* species, the conversion of substrates, and properties of extracted PHAs.

## 2. Metabolic Pathways and Key Enzymes Involved in the Synthesis of PHAs in *Aeromonas* spp.

**Figure 2:**
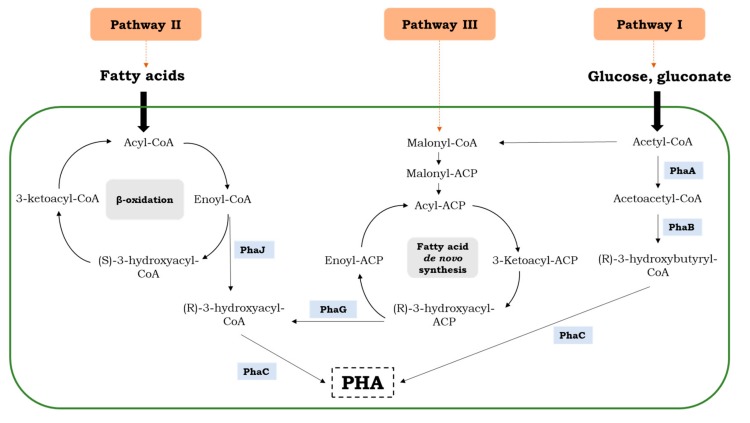
Metabolic pathways for synthesis of PHAs by the *Aeromonas* species.

The detailed knowledge of biosynthetic pathways of PHAs could be key to controlling this bioprocess and for producing PHAs with new properties. The metabolic pathway of PHA synthesis varies among microbial groups. So far, three pathways for the synthesis of PHA monomers in *Aeromonas* sp. have been well-studied. Pathway I utilizes sugars to generate the P(3HB) homopolymer, whereas copolymers are synthesized through pathways II and III (Figure 2).

It is well known that three key enzymes are responsible for PHA synthesis: β-ketothiolase, acetoacetyl-CoA reductase, and PHA synthase, encoded by the genes *phaA*, *phaB,* and *phaC*, respectively. It has been confirmed that the expression of these genes interact with each other and could be induced at nutrient limiting conditions to synthesize specific monomers and polymers of PHAs [10] Furthermore, P(3HB-co-HHx) biosynthesis genes consist of *phaP*, *phaC,* and *phaJ* genes, encoding phasin, PHA synthase, and (R)-specific enoyl-CoA hydratase, respectively [9]. The precursors for P(3HB-co-3HHx) co-polymer synthesis are supplied through the fatty acid β-oxidation pathway. Its accumulation is dependent on the synthase ability and the number of precursors supplied through the fatty acid β-oxidation pathway [12]. PHA synthase is the essential enzyme of PHA biosynthesis. It catalyzes the conversion of 3-hydroxyacyl-CoA into PHA. PHA synthase in *Aeromonas* sp. exhibits broad substrate ranges and determines the monomer composition of the synthesized biopolyesters [13].

Carbon sources, supplied to the culture medium, are metabolized differently in bacteria. Fatty acids are degraded by *Aeromonas* spp. via the β-oxidation process. In this pathway, acyl-CoA derived from fatty acids is degraded, resulting in the formation of enoyl-CoA intermediates encoded by the *phaJ* gene, which may be converted to (R)-3-hydroxyacyl-CoA by the (R)-specific enoyl-CoA hydratase and may be incorporated into a growing polyester chain by the function of PHA synthase [3]. Only four different amino acids were determined during the amino acid sequence analysis of the *phaJ* gene of *A. hydrophila* 4AK4 compared to *A. caviae* [14]. It was reported that the acyl-CoA dehydrogenase that catalyzes the dehydration reaction of acyl-CoA to enoyl-CoA is the rate-limiting enzyme in this pathway [15]. The over-expression of this enzyme could lead to more efficient PHA accumulation [12]. The glycolytic and pentose phosphate pathways are activated to generate acetyl-CoA and NADPH when sugars are utilized. Then, the two acetyl-CoA molecules are combined into acetoacetyl-CoA by β-ketothiolase (PhaA), and subsequently 3-hydroxybutyryl-CoA is generated by acetoacetyl-CoA dehydrogenase (PhaB) using NADPH as a cofactor, and finally is polymerized into PHA by PhaC polymerase [5].

## 3. Polyhydroxyalkanoates Synthesis by *Aeromonas* Species

The genus *Aeromonas* seems to have a promising capacity to synthesize and accumulate PHAs. The ability to produce a high content of biopolymers depends on carbon source, cultivation period, temperature, pH, and the presence of macro and microelements [16]. Some *Aeromonas* strains were able to produce PHAs in satisfactory amounts from simple carbon sources (Table 1).

Poly(3-hydroxybutyrate) (P(3HB)) is the most common polyhydroxyalkanoate in nature, that could be also accumulated by *Aeromonas* spp. Goa et al. [14] revealed that *A. hydrophila* ATCC 7966T, grown on dodecanoate, provided as the only carbon source, was able to synthesize homopolymer P(3HB) at a concentration of 11.2% of cell dry weight (CDW). According to Lee et al. [17] *A. hydrophila* is able to produce about 16.0% of P(3HB) using glucose under phosphorus limitation. In addition, the authors reported that when hexanoate, heptanoate, or octanoate were used, there was no accumulation of PHA. A higher P(3HB) concentration was achieved during the two-stage batch cultivation of *Aeromonas* sp. AC_03 using pure glycerol as the only carbon source [18]. This strain synthesizes the P(3HB) homopolymer at the level of 42%, with a biomass concentration of 2.3 g/L. Furthermore, it was revealed that some fatty acids could support the synthesis and accumulation of homopolymer poly(3-hydroxyvalerate) (P(3HV)). Shen et al. [19] conducted the *A. hydrophila* 4AK4 cultivations using various fatty acids, such as valeric acid, heptanoic acid, nonanoic acid, and undecanoic acid. Interestingly, the presence of the P(3HV) homopolymer was only detected when the mineral medium was supplemented with undecanoic acid. Other analyzed short-chain length fatty acids have been reported not to support cell growth due to their high toxicity. In the one-step and two-step shake flask growth process, the homopolymer content in *A. hydrophila* 4AK4 cells was similar and reached approximately 45.0% of P(3HV), with a cell dry weight value of 5.0 g/L.

Besides homopolymers, the cultivations of *Aeromonas* spp. towards biosynthesis of copolymers has been also carried out. The biosynthesis of copolymers has attracted much attention because they consist of 3HB as a constituent, along with other hydroxy acid (HA) units of chain lengths ranging from 3 to 14 carbon atoms, which thus possess better properties and processability than P(3HB) or the P(3HV) homopolymer itself. Therefore, many researchers reported that the bacteria belonging to the *Aeromonas* genus are capable of synthesizing P(3HB-co-3HHx) copolymers only when fatty acids are provided as substrates in the cultivation media. The accumulation of the above-mentioned copolymers by *A. hydrophila* grown on fatty acids has been studied in great depth by several researchers. *A. hydrophila* 4AK4 is the representative P(3HB-co-3HHx) producing strain due to its substrate specificity [20]. Chen et al. [10] reported that this bacterium is able to synthesize the P(3HB-co-3HHx) copolymer in a two-stage fed-batch large scale cultivation using glucose in the growth phase and lauric acid in the PHA production stage. The authors found that *A. hydrophila* 4AK4 synthesized 51.5% of the P(3HB-co-3HHx) copolymer with 86.5 mol% of 3HB monomer. A similar concentration of this copolymer (50.7% of CDW) was determined by Liu et al. [20] by applying lauric acid in the cultivation of the same strain. Also, a high P(3HB-co-3HHx) content in *A. hydrophila* 4AK4 cells has been reached by Quang et al. [21] using dodecanoate as the sole substrate into the bioreactor. However, the same strain cultured on co-substrates of dodecanoate and gluconate (weight ratio 1:1) was able to synthesize only 32% of P(3HB-co-3HHx) at a biomass concentration of 12 g/L. Lower values of these copolymers were reached using hexanoate (8.7% of CDW) and octanoate (13.5% of CDW) [22]. A significant amount of the P(3HB-co-3HHx) copolymer (68.2% of CDW) was synthesized by wild type *Aeromonas* sp. KC007 grown on lauric acid. Furthermore, it was observed that glucose was the only carbon source that supported the robust growth of this bacterium, but no significant PHA accumulation was determined (1.6% of CDW) [23].

**Table 1:** An overview of microbial synthesis of PHAs by *Aeromonas* sp. grown on different carbon sources.

Bacteria	Substrate	Environmental Stress	Biomass Concentration (g/L)	PHA Content (%)	Type of PHAs	References
*A. hydrophila*	glucose	without limitation	3.25	nd	nd	[17]
		P-limitation	1.90	15.6	P(3HB)	
		N-limitation	0.55	nd	nd	
	sodium gluconate	without limitation	3.05	nd	nd	
		P-limitation	4.63	35.1	P(3HB)	
		N-limitation	0.58	nd	nd	
	lauric acid	without limitation	7.68	19.5	P(3HB-co-HHx)	
		P-limitation	2.83	28.8	P(3HB-co-HHx)	
		N-limitation	1.70	22.5	P(3HB-co-HHx)	
*A. hydrophila* 4AK4	lauric acid and glucose	N-limitation	11.20	22.0	P(3HB-co-HHx)	[10]
		P-limitation	16.20	51.5	P(3HB-co-HHx)	
*A. hydrophila* 4AK4	dodecanoic acid	N-limitation	3.50	58.9	P(3HB-co-HHx)	[24]
*A. hydrophila* CGMCC 0911	lauric acid	N-limitation	2.24	49.0	P(3HB-co-HHx)	[12]
	oleic acid		0.52	43.0	P(3HB-co-HHx)	
*A. hydrophila* 4AK4	dodecanoic acid	without limitation	40.4	54.6	P(3HB-co-HHx)	[21]
	dodecanoic acid + gluconate		12.00	32.0	P(3HB-co-HHx)	
*Aeromonas* sp. KC 007	starch	N-limitation	1.74	1.8	P(3HB-co-HHx)	[23]
	lauric acid		1.32	68.2	P(3HB-co-HHx)	
*A. hydrophila* 4AK4	undecanoic acid	N-limitation	5.01	45.2	P(3HV)	[19]
*A. hydrophila* 4AK4	lauric acid and valeric acid (0.5 mL/L)	N-limitation	3.56	19.6	P(3HB-co-3HV-3HHx)	[25]
	lauric acid and valeric acid (4.0 mL/L)		1.18	41.4	P(3HB-co-3HV-3HHx)	
	lauric acid and valeric acid (5.0 mL/L)		1.15	35.1	P(3HB-co-3HV-3HHx)	
*A. hydrophila* 4AK4	lauric acid	N-limitation	4.85	40.7	P(3HB-co-HHx)	[22]
	hexanoate		0.61	8.8	P(3HB-co-HHx)	
	octanoate		1.18	13.5	P(3HB-co-HHx)	
*A. hydrophila* 4AK4	lauric acid	N-limitation	4.15	50.7	P(3HB-co-HHx)	[20]
*A. hydrophila* 4AK4	dodecanoate	N-limitation	4.73	49.7	P(3HB-co-HHx)	[14]
*A. hydrophila* 7966	dodecanoate	N-limitation	2.43	11.2	P(3HB)	
*A. hydrophila*	coconut oil	N-limitation	7.31	49.6	P(3HB)	[26]
*Aeromonas* sp. AC_03	pure glycerol	N-limitation	2.53	4.7	P(3HB)	[18]
		P-limitation	2.32	42.0	P(3HB)	
*Aeromonas* sp. AC_02	crude glycerol	N-limitation	2.00	2.8	P(3HB)	
		P-limitation	2.65	13.6	P(3HB)	

3HB—3-hydroxybutyrate; 3HV—3-hydroxyvalerate; 3HHx—3-hydroxyhexanoate; N-limitation—nitrogen limitation; P-limitation—phosphorus limitation; nd—not detected.

Wild type *A. hydrophila* 4AK has also been reported to synthesize terpolyesters containing 3HB, 3HV, and 3HHx monomers from a mixture of lauric acid and undecanoic acid [12]. However, the 3HV content was below 10%. When mixed carbon sources of lauric acid and valerate were applied, *A. hydrophila* 4AK synthesized P(3HB-co-3HV-co-3HHx) with higher 3HV content. Furthermore, the results showed that with increasing valerate concentration, biomass concentration decreased. However, the content of 3HB, 3HV, and 3HHx monomers in the terpolyesters remained relatively constant, at valerate concentrations ranging from 2 to 5 g/L [25].

The overall costs of polyhydroxyalkanoate production are higher compared to synthetic polymers. Therefore, attempts have been made to reduce the price of commercially produced PHAs by employing renewable carbon sources as reasonable and excellent potential feedstocks for industrial PHA production. Only starch and waste glycerol were tested for PHA synthesis by *Aeromonas* spp. Because of the higher levels of reduced carbon atoms in glycerol compared to carbohydrates, bacterial cells using glycerol are in a more reduced physiological state, stimulating intracellular polymer synthesis [27]. Crude glycerol, the main by-product from the biodiesel industry, was efficiently used by some strains of *Aeromonas* sp. According to Możejko-Ciesielska and Pokój [18], the AC_03 strain belonging to *Aeromonas* sp. is able to synthesize P(3HB) at a level of 22.1% of CDW when waste glycerol was supplied as the only carbon source. Furthermore, the authors confirmed that the ability to synthesize PHAs on crude glycerol is dependent on the bacterial strain. *Aeromonas* sp. AC_01 and AC_02 accumulated P(3HB) in a lesser concentration in comparison to the AC_03 strain; the maximum PHA content reached the values of 11.1% and 13.6%, respectively. Also, starch. as one of the most important products synthesized by plants, was used as an alternative substrate for PHA production by *Aeromonas* sp. KC 007 [23]. However, even though this bacterium grew efficiently on this substrate, the P(3HB-co-3HHx) copolymer was accumulated in trace amounts (1.8% of CDW).

It is well known that bacteria accumulate PHAs to store carbon and energy when environmental conditions are disturbed. Interestingly, the data from literature confirmed that the PHA content in *Aeromonas* sp. cells is higher under a phosphorus-limiting environment compared to nitrogen starvation. Lee et al. [28] revealed that *A. hydrophila* accumulated a P(3HB) homopolymer grown on glucose or sodium gluconate only under phosphorus limitation. Furthermore, when fatty acids were used as carbon sources, the highest PHA content was determined under the phosphorus-limiting condition. The same observations were made by Chen et al. [10], who described that PHA content in *A. hydrophila* 4AK4 cells cultured under phosphorus limitation was 2.3 times higher (51.5% of CDW) than that obtained under nitrogen limitation (22.0% of CDW). It was observed that nutrient limitation is more favorable for the synthesis of PHAs in the *Aeromonas* sp. AC_01, AC_02, and AC_03 strains grown on pure and crude glycerol [18]. The data indicate that biopolymer concentration was higher under phosphorus starvation, except for the AC_03 strain grown on crude glycerol during one-stage cultivation, when three times more biopolymers were synthesized and accumulated under nitrogen-limiting conditions. However, the highest PHA concentration was extracted during the two-step bioprocess of *Aeromonas* spp. AC_03, cultured on pure glycerol (42% of CDW) under phosphorus limitation.

## 4. PHA Synthesis by Genetically Modified *Aeromonas* Species

In addition to the wild strains, efforts have been made to use genetically modified *Aeromonas* strains for enhanced PHA biosynthesis (Table 2). In particular, *A. hydrophila* 4AK4, as the best-studied strain, has been engineered to improve PHA productivity. Its properties, such as robust growth, simple growth requirements, and convenience for genetic engineering, make this strain an ideal host for PHA biosynthesis [29].

Shen et al. [19], working with *A. hydrophila* 4AK4 harboring *vgb* encoding *Vitreoscilla* haemoglobin and *fadD* encoding *Escherichia coli* acyl-CoA synthase, successfully produced the homopolymer P(3HV). The authors suggested that the over-expression of *vgb* and *fadD* genes in recombinant *A. hydrophila* 4AK4 positively influenced PHA concentration. In the one-stage and two-stage shake flask growth process, the PHA content of this bioengineered bacterium was higher in comparison to that of the wild type, and reached the maximum concentration of 47.7% of CDW. Han et al. [24] demonstrated that *A. hydrophila* 4AK4 harboring *phaP, phaC, phaJ, phaPC, phaCJ*, and *phaJP* genes from *Aeromonas punctata* can produce P(3HB-co-3HHx) on dodecanoate. The authors observed that the expression of the *phaC* gene led to an increase of 3HHx fraction from 14 mol% to 23 mol%. Furthermore, the expression of *phaC* and *phaJ* genes enhanced the copolymer synthesis up to 64% of CDW. Furthermore, the ability of *A. hydrophila* CQ4 to produce random copolymers of P(3HB-co-3HA) was evaluated by Qin et al. [9]. The co-expression of (R)-specific enoyl-CoA hydratase and PHA synthase (PhaC2) from *Pseudomonas stutzeri* 1317 enabled *A. hydrophila* CQ4, synthesizing 20.86% of this copolyester containing 59 mol% 3HB, 37 mol% 3HHx, and trace amounts of 3HO, 3HD, and 3HDD. Moreover, recombinant *A. hydrophila* 4AK4, harboring *phaC1* from *Pseudomonas stutzeri* 1317, *fadD* encoding *Escherichia coli* acyl-CoA synthase and *fadL* from *Pseudomonas putida* KT2442, with its native *pha* synthase gene deleted, was reported to accumulate up to 54% of poly(3HB-co-3HHx) and 51% poly(3HB-co-3HHx-co-3HO) during a two-step cultivation process under nitrogen limitation, when grown on sodium hexanoate or sodium octanoate, respectively. In addition, this recombinant strain produced 3HB, 3HHx, 3HO, and 3HDD, with 3HHx sharing the largest content of 67 mol% grown on lauric acid [22]. Zhao and Chen [30] evaluated the capability of modified *A. hydrophila* 4AK4, harboring *phbA* and *phbB* genes, encoding β-ketothiolase and the acetoacetyl-CoA reductase of *Ralstonia eutropha,* to accumulate the P(3HB-co-3HV-co-3HHx) terpolyester from mixtures of dodecanoic acid and propionic acid. Depending on the concentration of propionic acid in bacterial cultures, the terpolyester content in the *A. hydrophila* mutant ranged from 20.7% to 35.6% of its CDW. The same terpolyesters were reported to be synthesized by recombinant *A. hydrophila,* containing PHA synthesis genes *phaP*, *phaC,* and *phaJ,* and encoding the PHA binding protein phasin, PHA synthase, and enoyl-CoA hydratase, respectively [25]. Zhang et al. [25] revealed that the 3HV content in the terpolyester P(3HB-co-3HV-co-3HHx) could be successfully manipulated when the above-mentioned recombinant was grown on valerate. The authors proved that the mutant synthesized up to 71% of P(3HB-co-3HV-co-3HHx) terpolyester when fed with 2 g/L valerate, and additional feeding on valerate led to the reduction in PHA concentration in bacterial cells. It was also reported that the recombinant *A. hydrophila* 4AK4, harboring PHA synthesis genes *phaPCJ* cloned from *Aeromonas caviae,* synthesized the P(3HB-co-4HB-co-3HHx) terpolymer, with the monomer content of 4HB dependent on the concentration of 1,4-butanediol during growth with the supplementation of lauric acid [31].

**Table 2:** PHAs production by genetically modified *Aeromonas* spp.

Genetically Modified Bacteria	Gene Donor	Carbon Source	Biomass Concentration (g/L)	PHA Content (%)	Type of PHAs	References
*A. hydrophila* 4AK4 (harboring *phaJ* gene)	*Aeromonas punctata*	dodecanoate	3.7	51.6	P(3HB-co-3HHx)	[24]
*A. hydrophila* 4AK4 (harboring *phaP* gene)			4.4	57.8	P(3HB-co-3HHx	
*A. hydrophila* 4AK4 (harboring *phaC* gene)			4.3	58.9	P(3HB-co-3HHx)	
*A. hydrophila* CGMCC 0911	*Escherichia coli* JM 109	lauric acid	3.2	47.3	P(3HB-co-3HHx)	[12]
*A. hydrophila* 4AK4	*Escherichia coli* S17-1	gluconate	6.9	13.7	P(3HB-co-3HHx)	[32]
*A. hydrophila* 4AK4	*Aeromonas caviae*	dodecanoate	51.5	62.0	P(3HB-co-3HHx)	[21]
		dodecanoate + sodium gluconate	32.8	52.0	P(3HB-co-3HHx)	
*A. hydrophila* pBBR1MCS-2	*Escherichia coli* S17-1	lauric acid	4.9	49.9	P(3HB-co-3HHx)	[33]
*A. hydrophila* pBBRC	*Escherichia coli* S17-1	lauric acid	5.1	53.4	P(3HB-co-3HHx)	
*A. hydrophila CQ4*	*Pseudomonas stutzeri 1317*	dodecanoate + gluconate	2.5	2.9	P(3HHx-co-3HO-co-3HD-co-3HDD)	[9]
	*Pseudomonas stutzeri 1317* *Ralstonia eutropha*	dodecanoate + gluconate	3.6	20.9	P(3HB-co-3HHx-co-3HO-co-3HD-co-3HDD)	
*A. hydrophila* 4AK4	*Ralstonia eutropha*	dodecanoate + propionate	3.3	35.6	P(3HB-co-3HV-co-3HHx)	[30]
*Aeromonas* KC007-R1	*Cupriavidus necator* H16	glucose	1.3	36.8	P(3HB-co-3HHx)	[23]
		starch	1.8	32.7	P(3HB-co-3HHx)	
		lauric acid	1.6	59.4	P(3HB-co-3HHx)	
*A. hydrophila* 4AK4	*Aeromonas caviae*	lauric acid	4.8	58.6	P(3HB-co-3HHx)	[31]
		lauric acid + 1,4-butanediol	4.0	24.2	P(3HB-co-4HB-co-3HHx)	
*A. hydrophila* 4AK4	*Escherichia coli* *Vitreoscilla sp.*	undecanoate	5.6	47.7	P(3HV)	[19]
*A. hydrophila* 4AK4	*Aeromonas caviae*	valerate 0.5 g/L	3.7	45.4	P(3HB-co-3HV-co-3HHx)	[25]
		valerate 1 g/L	4.7	59.3	P(3HB-co-3HV-co-3HHx)	
		valerate 2 g/L	2.4	71.1	P(3HB-co-3HV-co-3HHx)	
*A. hydrophila* AKJ1	*Escherichia coli* *Pseudomonas stutzeri* *Pseudomonas putida KT2442*	sodium hexanoate	3.0	54.5	P(3HB-co-3HHx)	[22]
		sodium octanoate	3.1	50.6	P(3HB-co-3HHx-co-3HO)	

3HB—3-hydroxybutyrate; 4HB—4-hydroxybutyrate; 3HV—3-hydroxyvalerate; 3HHx—3-hydroxyhexanoate; 3HO—3-hydroxyoctanoate.

## 5. Properties of PHAs Produced by *Aeromonas*

Polyhydroxyalkanoates synthesized by wild type and recombinant bacteria can differ in chemical, physical, and mechanical features. They have similar properties to petrochemical-based polymers like polypropylene. The PHA properties depend on the biopolymer producing strain, fermentation conditions, and carbon source used. A detailed overview of PHA properties synthesized by *Aeromonas* spp. is presented in Table 3.

It is known that scl-PHAs are brittle, stiff, and possess high crystallinity, high melting and low glass transition temperatures. Mechanical properties, such as the Young’s modulus and the tensile strength, are very similar to those of polypropylene. However, the extension to break for P(3HB) is much lower in comparison to synthetic polymers. Much better properties were reported for scl-copolymers. They have much lower melting points than scl-homopolymers, making them better polymers for in-service applications of PHAs. Moreover, they are less crystalline, easier to mold, and tougher [34]. In addition, these thermomechanical properties can be widely varied by the proportion of the constituent monomer units [35]. On the other hand, mcl-PHAs have more advantageous properties than scl-PHAs. They are thermo-elastomers with a low crystallinity, a low melting temperature, ranging between 40 and 86 °C, a low tensile strength, and a high elongation to break. Furthermore, the molecular weight of polyhydroxyalkanoates is regarded as a parameter affecting their properties. Number (M_n_) and weight (M_w_) average molecular weights of mcl-PHAs are lower in comparison to those of scl-PHAs.

Shen et al. [19] reported that the M_w_ and M_n_ for the P(3HV) homopolymer synthesized by recombinant *A. hydrophila* 4AK4 grown on undecanoic acid were 230,000 and 65,340 Da, respectively. The polydispersity index (I = M_w_/M_n_) bore a large value of 3.52. The authors observed that the thermal properties of extracted P(3HV) with ΔH_m_ = 47 J/g were better than those of P(3HB), suggesting that P(3HV) has a wider processing window. The P(3HB-co-3HHx) copolymer extracted from *A. hydrophila* AKJ1 harboring *phaC1ps*, *fadDec* and *fadLpp* possessed a higher M_w_ value (300,000 Da). This copolymer was reported as a sticky amorphous polymer without a remarkable T_m_ value, with low T_g_ (−20.8 °C) and a decomposition temperature similar to the P(3HV) homopolymer [22].

Moreover, physical properties of the terpolyesters extracted and purified from recombinant *Aeromonas* sp. changed with variations in the monomer composition. Xie and Chen [31] demonstrated that the introduction of 4HB and 3HHx monomers into P(3HB) enhanced its thermal stability, and changed its crystallinity and flexibility compared with the homopolyester P(3HB). The presence of 4.3–6.6 mol% 4HB fractions, together with 22–19.5 mol% 3HHx, in these terpolyesters led to a higher value of elongation to breaks. The Young’s modulus and tensile strength of the terpolyesters were lower compared to P(3HB). The introduction of the 4HB monomer improved the flexibility of the P(3HB-co-4HB-co-3HHx) terpolymer. Moreover, the results suggested that the extracted P(3HB-co-3HV-co-3HHx) terpolyesters with different monomer contents showed varied thermal and mechanical properties. Those with higher 3HV fractions revealed an improved property over the lower HV containing ones [25]. The authors confirmed that terpolyesters P(79% 3HB-co-11% 3HV-co-10% 3HHx) and P(73% 3HB-co-17% 3HV-co-10% 3HHx) reached a Young’s modulus, elongation to break, and tensile strength ranging from 97 to 235 Mpa, 341% to 833%, and 8.4 to 14.3 Mpa, respectively. Furthermore, increasing the fraction of 3HHx contents in the terpolyesters from 10.7 mol% to 13.4 mol% improved the elongation at break from 276.9% to 481.1% and Young’s modulus was reduced from 318.9 MPa to 109.8 MPa [30]. Moreover, the authors suggested that the introduction of 3HV and 3HHx constituents with soft and non-crystal properties provided the material with higher rigidity and higher ductility, respectively.

**Table 3:** Comparison of material properties of PHAs extracted from *Aeromonas* sp. cells and synthetic polymers.

Bacteria	Type of PHAs/Synthetic Polymers	Molecular Weight (*M*_w_ × 10^4^ Da)	*T*_g_ (°C)	*T_d_* (°C)	*T_m_* (°C)	Young’s Modulus (MPa)	Elongation to Break (%)	Tensile Strength (Mpa)	Biodegradation	References
Recombinant *A. hydrophila* 4AK4	P(84.7% 3HB-co-5.4% 3HV-co-9.9% 3HHx	37.3	−2.6	273.1	129	nd	nd	nd	high rate	[30]
	P(83.7% 3HB-co-1.2% 3HV-co-15.1% 3HHx)	52.8	−1.8	248.5	104	nd	nd	nd		
	P(88.3% 3HB-co-1.0% 3HV-co-10.7% 3HHx)	nd	nd	nd	nd	318.9	276.9	10.1		
	P(84.2% 3HB-co-2.4% 3HV-co-13.4% 3HHx)	nd	nd	nd	nd	109.8	481.1	8.0		
Recombinant *A. hydrophila* 4AK4	P(73.7% 3HB-co- 4.3% 4HB-co- 22% 3HHx)	66.6	−9.3	253	nd	3.8	504	0.6	high rate	[31]
	P(73.8% 3HB-co-7.6% 4HB-co-18.6% 3HHx)	75.4	−11.7	245	nd	2.85	143	0.3		
Recombinant *A. hydrophila* 4AK4	P(3HV)	23.0	−15.8	258	103	nd	nd	nd	high rate	[19]
Recombinant *A. hydrophila* 4AK4	P(75.3% 3HB-co-13.1% 3HV-co-11.7% 3HHx)	94.4	−1.8	248.1	101.3	nd	nd	nd	high rate	[25]
	P(47.9% 3HB-co-23.8% 3HV-co-28.3% 3HHx)	94.2	−5.1	250.5	54.2	nd	nd	nd		
	P(78.8% 3HB-co-10.9% 3HV-co-10.3% 3HHx)	nd	nd	nd	nd	234.9	340.9	8.4		
	P(55.2% 3HB-co-25.7% 3HV-co-19.1% 3HHx)	nd	nd	nd	nd	2.08	133.3	0.3		
Recombinant *A. hydrophila* AKJ1	P(4% 3HB-co-96% 3HHx)	30.0	−20.8	257	nd	nd	nd	nd	high rate	[22]
	P(3% 3HB-co-87% 3HHx-co-10% 3HO)	16.0	−23.2	264	nd	nd	nd	nd		
**Synthetic polymers**										
	Polypropylene	nd	−10	nd	176	1.7	400	34.5	slow rate	[36]
	Polystyrene	nd	100	nd	240	3.1	nd	50		
	Polyethylene	nd	−30	nd	130	0.2	620	10		[37]

3HB—3-hydroxybutyrate; 4HB—4-hydroxybutyrate; 3HV—3-hydroxyvalerate; 3HHx—3-hydroxyhexanoate; 3HO—3-hydroxyoctanoate; M_w_—weight-average molecular weight; T_g_—glass-transition temperature; T_m_—melting temperature; T_d_—Temperature at 5% weight loss was determined by TGA; nd—not detected.

## 6. Conclusions and Future Prospects

Due to their biodegradability, biocompatibility, and nontoxicity, polyhydroxyalkanoates are useful and valuable materials that can be used in many industrial fields, ranging from everyday products to medical applications. They are considered the most promising alternative to petroleum-based plastics for the future. One of the major problems in the commercialization of PHAs is still the high manufacturing cost. Therefore, much effort has been devoted to the development of efficient fermentation with different bacterial species. Within 30 years of research on PHAs, great progress has been made regarding their biosynthesis, and metabolic and genetic regulation. Many bacteria have been reported to be able to synthesize PHAs, but *Aeromonas* spp. seems to be a potential cell factory for the production of these biopolyesters.

**Figure 3:**
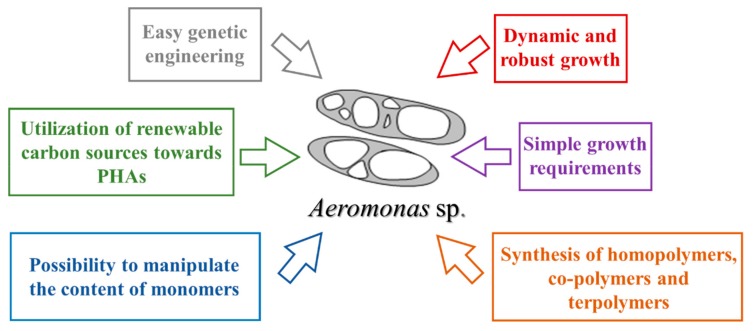
Advantages of using *Aeromonas* spp. for large scale production of polyhydroxyalkanoates.

There are a number of advantages of using *Aeromonas* spp. for the synthesis of polyhydroxyalkanoates (Figure 3). These bacteria are characterized by robust growth, simple growth requirements, the ability for the synthesis of homopolymers, co-polymers, and terpolymers with unique material properties, making them ideal hosts for the industrial production of biopolyesters. High-cell-density cultivation of *A. hydrophila* has been demonstrated previously, giving hope that this bacterium can be a suitable candidate for the industrial production of PHAs [10,17]. To date, only a few studies have been carried out so far regarding the improvement of *Aeromonas* spp. biomass concentration, PHAs accumulation rate, and their properties. The genetic engineering of biopolyester producers belonging to the *Aeromonas* species has enabled the improvement of PHA yields. However, it is essential to have a deeper understanding of genetic and metabolic regulation that could lead to the construction of a bacterial host being capable of providing a consistent monomer composition and ensuring constant material properties that are essential for their applicability.

The above-mentioned data confirm that not too many attempts have been made to reduce the price of PHAs produced by *Aeromonas* spp. by using cheap carbon sources. It is known that wild type *Aeromonas* spp. are unable to utilize carbohydrates, such as starch, to accumulate PHA, which has definitely hampered the use of this bacterium in applications for industrial-scale PHA production [23]. However, many other renewable carbon sources, like waste from the food industry, should be taken into account as possible, cheap substrates used for the growth of *Aeromonas* spp. to make the whole bioprocess more feasible. The limited knowledge of the PHA central biochemical network when using low-cost carbon sources limits the use of *Aeromonas* spp. in such endeavors. Therefore, it is essential to pay much attention to the high-throughput screening of potentially new bacterial strains and to optimize the fermentation process using renewable carbon sources. Also, engineering the bacteria belonging to this genus for the efficient utilization of waste substrates could expand the spectrum of applicable carbon sources and make this bioprocess more feasible. Since the complete genome of *A. hydrophila* 4AK4 was sequenced and analyzed, it provided many possibilities for metabolic engineering [14]. There is a need for an “omics” analysis that could help provide more understanding of metabolic pathways and would be helpful for improving PHA productivity.
